# In Vitro Models in Chronic Obstructive Pulmonary Disease (COPD): Implications for New Diagnostic Strategies and Therapeutic Approaches

**DOI:** 10.3390/biology15141104

**Published:** 2026-07-08

**Authors:** Gioacchin Iannolo, Rosaria Tinnirello, Valentina Lazzara, Bruno Douradinha, Vitale Miceli, Giusy Daniela Albano

**Affiliations:** 1 IRCCS ISMETT (Istituto Mediterraneo per i Trapianti e Terapie ad Alta Specializzazione), Via E. Tricomi 5, 90127 Palermo, Italy; rtinnirello@ismett.edu (R.T.); vmiceli@ismett.edu (V.M.); 2 Department of Medicine and Surgery, Università degli Studi di Enna “Kore”, 94100 Enna, Italy; 3 Department of Economics, Business and Statistics (dSEAS), University of Palermo (UNIPA), 90128 Palermo, Italy; valentina.lazzara@unipa.it; 4 Institute of Translational Pharmacology (IFT), National Research Council (CNR), Via Ugo La Malfa 153, 90146 Palermo, Italy; 5 OneCapsule, Marousi, 15126 Athens, Greece

**Keywords:** COPD, air-liquid interface (ALI), lung organoids, lung-on-a-chip, precision-cut lung slices (PCLS), lung ECM-derived hydrogels, extracellular vesicles, microRNAs, organ failure, personalized medicine

## Abstract

Chronic obstructive pulmonary disease (COPD) represents a major global health issue, characterized by persistent airflow limitation, chronic inflammation, and progressive tissue remodeling. Its clinical and molecular heterogeneity, combined with the lack of resolutive therapies, underscores the urgent need for advanced experimental tools to improve understanding and therapeutic development. Traditional 2D cell culture systems, though historically useful, fail to replicate the complexity of the human lung. In this review, we analyze the remarkable relevance of advanced 3D models for studying COPD pathophysiology, including epithelial injury and regeneration, extracellular matrix remodeling, and interactions with environmental triggers such as cigarette smoke and airborne pollutants. Three-dimensional in vitro models, such as ALI cultures, lung organoids, and lung-on-a-chip platforms, PCLS, and lung ECM-derived hydrogels offer more physiologically relevant environments to investigate epithelial dysfunction, immune responses, and host-pathogen interactions. We discuss the contribution of viral and bacterial infections to COPD exacerbations, and explore how 3D models have become essential tools for modeling these events. We also highlight recent advances in personalized medicine that use patient-derived organoids and ALI cultures for drug screening and biomarker discovery. Furthermore, we examine the therapeutic potential of probiotics and extracellular vesicle-associated microRNAs to modulate inflammation and epithelial repair. Collectively, these innovative systems represent powerful platforms to promote precision medicine in COPD.

## Simple Summary

Chronic obstructive pulmonary disease (COPD) represents a major global health burden, prompting substantial efforts to identify and promote novel therapeutic strategies. However, the physiological relevance of conventional two-dimensional cell culture systems often limits the effectiveness of high-throughput screening approaches. This review explores three-dimensional models that better reproduce key structural and functional features of this complex, multifactorial disease. Particular attention is given to their application in evaluating emerging therapeutic approaches, including probiotics and extracellular vesicle-associated microRNAs.

## 1. Introduction

Chronic obstructive pulmonary disease (COPD) is a major global health challenge that significantly impacts health care systems worldwide. With an estimated 300 million people suffering from it, COPD is the fourth leading cause of death globally [1,2,3]. COPD is characterized by irreversible airflow limitation and progressive respiratory symptoms, including chronic cough, mucus production, and dyspnea, which worsen over time.

COPD manifests itself in various forms, including emphysema, chronic bronchitis, and small airway disease, with overlapping features between these subtypes [4]. This heterogeneity is one of the key challenges in understanding the pathophysiology of COPD and developing effective treatments. This disease affects the lung parenchyma and airways in different ways, with variable degrees of tissue damage, airway remodeling, and functional decline. While chronic bronchitis is characterized by airway inflammation and mucus hypersecretion [5], emphysema is defined by the destruction of alveolar walls and the formation of enlarged airspaces, resulting in impaired gas exchange [6]. Additionally, some COPD patients exhibit features of asthma, a condition that further complicates diagnosis and management [7]. At the cellular and molecular level, COPD is driven by chronic inflammation in the airways, alveoli, and vasculature. Inhalation of harmful substances such as cigarette smoke, air pollutants, occupational dust, and fumes [8,9] triggers the innate immune system. This leads to the recruitment of neutrophils, macrophages, and T lymphocytes. These immune cells release a wide range of pro-inflammatory mediators, including cytokines (e.g., tumor necrosis factor alpha [TNF-α]), chemokines (e.g., interleukin-8 [IL-8]), and proteases such as matrix metalloproteinases (MMPs) that play a crucial role in tissue destruction, mucus production, and fibrosis [10,11,12,13]. Chronic inflammation drives airway remodeling, fibrosis, and the progressive destruction of the lung parenchyma, particularly in the alveoli. The result is the characteristic loss of lung function observed in COPD patients [14]. The airway epithelium plays a pivotal role in initiating and maintaining the inflammatory response in COPD. Under normal conditions, the epithelium acts as a protective barrier against inhaled pathogens, particulates, and pollutants. However, in COPD, repeated injury from these harmful substances leads to impaired epithelial repair and regeneration. The damaged epithelium is unable to fully restore its integrity, leading to chronic inflammation and tissue remodeling. This impaired repair capacity is one of the main features of COPD pathogenesis, and contributes to the progressive nature of the disease [15,16]. Furthermore, respiratory pathogens play a role in disease progression and acute exacerbations [17,18]. Recent evidence indicates that the dysregulation of extracellular vesicles (EVs), along with oxidative stress, epigenetic modifications, and microRNAs (miRNAs), constitute critical factors able to influence COPD pathogenesis and progression [19].

An important limitation in COPD research is the lack of robust in vitro models that recapitulate the cellular complexity and dynamic environment of the human lung. Conventional two-dimensional (2D) culture systems are unable to simulate spatial architecture, mechanical forces, and multicellular interactions that occur in vivo [20]. Consequently, the integration of advanced three-dimensional (3D) platforms is essential to support translational studies and enhance the relevance of preclinical findings [21]. The development of such models requires systematic efforts in model validation, cell sourcing, and integration of patient-derived materials, as emphasized by recent expert roadmaps [22].

Despite their many limitations, traditional 2D cell culture models are instrumental in studying the molecular mechanisms behind COPD. These models typically consist of a single layer of epithelial cells cultured on a flat surface, which fails to accurately represent the complex 3D structure of the lung [23,24]. Indeed, 2D culture cells are exposed to the same conditions across the entire monolayer, and the lack of architectural complexity implies that key interactions between different cell types and the extracellular matrix (ECM) are not fully assessed. In contrast, 3D culture systems provide a more physiologically relevant environment for studying the pathophysiology of COPD [25]. By simulating the architecture of the lung and the interactions between epithelial cells, immune cells, and ECM components, 3D models offer a more accurate representation of the cellular responses to injury and inflammation [25,26]. These models enable the study of tissue remodeling, epithelial dysfunction, and barrier integrity, all of which are critical aspects of COPD progression. Additionally, 3D models can be used to investigate how chronic exposure to harmful substances, such as cigarette smoke or pollutants, impacts epithelial repair and regeneration, providing valuable insights into the mechanisms driving disease progression [27,28].

Furthermore, current strategies promote the use of multi-cellular and patient-specific models to capture heterogeneity in disease mechanisms and therapy responses. For instance, the use of induced pluripotent stem cell (iPSC)-derived epithelial cells or primary cells obtained from well-phenotyped COPD patients, can support personalized approaches and improve biomarker identification for precision medicine applications [22,29].

The integration of air-liquid interface (ALI) cultures into 3D models has further enhanced their relevance in COPD research. ALI cultures are particularly valuable for studying lung diseases such as COPD, allowing researchers to investigate the effects of air exposure and inhaled pollutants on epithelial cell function and barrier integrity [23]. Furthermore, ALI cultures can be combined with other advanced 3D models, such as lung organoids or lung-on-a-chip systems, to create more complex and dynamic platforms to study COPD pathophysiology [25,26]. The application of 3D lung models has also expanded to studying COPD exacerbations triggered by viral and bacterial infections. By using ALI cultures and lung-on-a-chip systems, researchers can investigate host-pathogen interactions in a physiologically relevant setting, facilitating the development of targeted antiviral and antibacterial strategies [30,31].

The most advanced approaches aim to replicate both the cellular and biomechanical properties of the lung, including air pressure, cyclic stretch, and microvascular flow. These features are particularly evident in lung-on-a-chip systems [32,33]. Such dynamic platforms improve the assessment of epithelial repair kinetics, drug responsiveness, and pathogen entry mechanisms under conditions that simulate physiological or pathological stress, including exacerbations or hypoxia [32,34,35].

In this review, we aim to critically evaluate the potential of advanced 3D culture models, such as ALI cultures, organoids, and lung-on-a-chip platforms, and elucidate complex cellular and molecular mechanisms leading to COPD pathology. Specifically, we explore how these models contribute to the investigation of epithelial dysfunction, chronic inflammation, and host-pathogen interactions, particularly during disease exacerbations triggered by viral and bacterial infections. Furthermore, we examine the role of 3D systems in personalized medicine and drug discovery. Finally, we highlight the diagnostic and therapeutic potential of extracellular vesicles (EVs) and microRNAs (miRNAs) in COPD, proposing their integration with 3D platforms to enhance biomarker identification, and support the development of innovative, targeted treatment strategies. While several reviews have addressed the use of 3D lung models in respiratory diseases, this review combines three key aspects: a critical, side-by-side comparison of ALI cultures, organoids, lung-on-a-chip, PCLS and ECM-derived hydrogels in COPD; how these models connect with EV- and miRNA-based diagnostics and therapeutics; and probiotic strategies as emerging candidates for testing in 3D co-culture. Our aim is to bridge reductionist in vitro findings to patient-centered clinical applications, with particular attention to the trends currently reshaping the field: patient-specific disease modelling, single-cell and spatial methods, AI-assisted analysis, and multi-omics integration.

## 2. COPD Cellular Models

**Figure 1:**
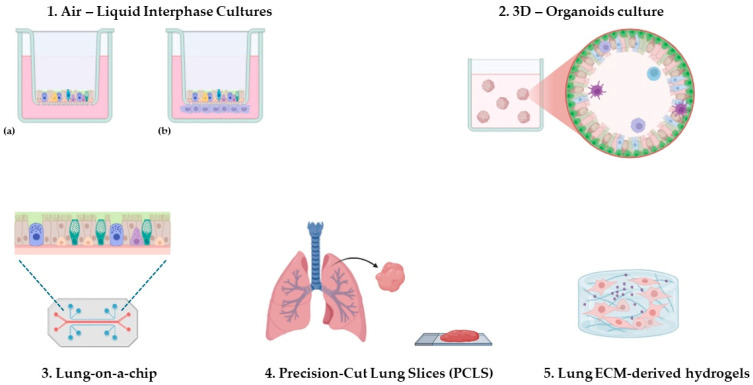
Schematic overview of advanced 3D in vitro lung models for COPD research. 1. Air–Liquid Interface (ALI) Cultures: (**a**) Airway epithelial cells are cultured on permeable membranes (synthetic or biological) with the apical surface exposed to air and the basal surface in contact with the culture medium. This configuration has shown to promote a range of cellular processes, including epithelial polarization, mucociliary differentiation, and the formation of a functional barrier. (**b**) Epithelial cells can also be co-cultured with additional cell types, such as fibroblasts (in the basal compartment), endothelial cells (on the opposite side of the membrane), and immune cells (e.g., macrophages or dendritic cells added to the apical or basal compartments). This allows to model epithelial–mesenchymal and immune interactions more realistically. 2. 3D Lung Organoids: Self-organizing spherical structures derived from primary airway basal cells or pluripotent stem cells, embedded in extracellular matrix-based hydrogels (e.g., Matrigel). These organoids recapitulate key aspects of epithelial tissue architecture, multilineage differentiation, and regenerative potential. 3. Lung-on-a-chip Devices: Microfluidic platforms composed of parallel microchannels lined with epithelial and endothelial cells, separated by a porous membrane. These systems are designed to simulate key biomechanical and biochemical features of the lung, including air flow, vascular perfusion, and cyclic mechanical stretch. 4. Precision-Cut Lung Slices (PCLS): Thin slices of intact lung tissue (typically 100–300 µm) preserving native airway and alveolar architecture, and containing epithelial, endothelial, mesenchymal, and immune cell populations. PCLS facilitate the execution of functional ex vivo studies within a physiologically relevant multicellular microenvironment. 5. Lung ECM-Derived Hydrogels: Decellularized lung extracellular matrix processed into hydrogels that retain native biochemical cues. Various cell types, including epithelial, stromal, and immune cells, can be embedded to study cell–matrix interactions, remodelling, and morphogenesis in a 3D context.

The human lung is a highly complex organ composed of conducting airways and alveolar regions, lined by a pseudostratified epithelium in the upper airways and a simple squamous epithelium in the alveoli [36]. Alveolar sacs, consisting of type I and type II pneumocytes, are critical for gas exchange and surfactant production. Basal, club, goblet, and ciliated epithelial cells contribute to mucociliary clearance and innate immunity within the bronchial tree [37]. Lung structural and cellular heterogeneity plays a fundamental role in its homeostasis and immune response to injury. Chronic obstructive pulmonary disease (COPD) disrupts this architecture through a combination of airway inflammation, epithelial damage, mucus hypersecretion, and alveolar destruction (emphysema), often triggered by exposure to cigarette smoke or environmental pollutants [38]. This multifaceted pathophysiology requires in vitro models capable of recapitulating both the cellular complexity and the 3D organization of the lung microenvironment. Traditional 2D monolayer cultures lack spatial orientation, mechanical cues, and multicellular interactions observed in vivo. In contrast, 3D models (including ALI systems, lung organoids, PCLS, and lung-on-a-chip platforms) offer enhanced physiological relevance incorporating relevant lung-resident cell types such as epithelial cells (e.g., basal, ciliated, goblet), fibroblasts, endothelial cells, and immune cells [39]. These systems enable to study tissue remodeling, epithelial barrier dysfunction, immune responses, and drug permeability under conditions that closely mimic the in vivo lung. From a therapeutic perspective, the current clinical management of COPD includes bronchodilators (β2-agonists and muscarinic antagonists), corticosteroids, and phosphodiesterase-4 inhibitors. In parallel, ongoing clinical trials are exploring biologic therapies that target inflammatory pathways [40]. However, the heterogeneity of COPD and its variable response to treatment require predictive and personalized in vitro models supporting drug screening and mechanistic studies [41]. A wide array of in vitro and ex vivo models has been developed to investigate the cellular and molecular mechanisms of COPD, each with distinct advantages and limitations depending on the research question [42,43,44]. Among these, ALI cultures, 3D organoid systems, lung-on-a-chip technologies, PCLS, and lung ECM-derived hydrogels are increasingly used to replicate the complex pathophysiology of COPD [24,45]. Despite traditional 2D models are still widely used for basic mechanistic studies, these advanced platforms offer improved physiological relevance by reproducing the multicellular architecture, mechanical dynamics, and cell–cell interactions of the human lung. ALI cultures allow epithelial cells to differentiate into functionally distinct subtypes at the interface between air and medium, simulating the bronchial epithelium under exposure to air pollutants [46]. Three-dimensional organoid cultures can recreate the spatial structure and multilineage composition of airway or alveolar regions, while co-culture formats allow the investigation of epithelial–stromal or epithelial–immune interactions [47]. Lung-on-a-chip systems, through their microfluidic design, enable dynamic modeling of airflow, stretch, and blood flow, mimicking essential aspects of lung physiology [32]. PCLS maintain native tissue architecture and include all resident cell types and ECM components, offering a high-fidelity ex vivo platform to study airway inflammation and remodeling [48,49]. Lung ECM-derived hydrogels, obtained from decellularized lung tissue, provide a bioactive and tunable scaffold that recapitulates the biochemical and mechanical properties of the lung microenvironment [49]. These hydrogels enable 3D cultures of epithelial and stromal cells within disease-specific ECM contexts, facilitating studies of COPD-associated fibrosis, epithelial dysfunction, and therapeutic response [50,51]. A schematic representation of these advanced 3D in vitro lung models is shown in Figure 1.

In the following sections, we describe in detail the application, relevance, and limitations of the models for the study of COPD pathogenesis, focusing on epithelial dysfunction, inflammation, repair mechanisms, and exacerbation biology. Because these platforms are not interchangeable, we compare using criteria that typically guide model selection. These include physiological fidelity, the presence of mechanical and vascular cues, throughput and scalability, reproducibility and standardization of cell sources, achievable culture duration, and translational readiness. ALI cultures and organoids are accessible and support patient-specific differentiation, but they lack vascular and mechanical components. Lung-on-a-chip systems restore biomechanical and barrier dynamics, albeit at the expense of throughput and technical complexity. PCLS preserve native multicellular architecture but have limited viability and high donor variability, while ECM-derived hydrogels reproduce disease-specific matrix cues yet continue to face challenges in standardization (Table 1).

### 2.1. Air-Liquid Interface (ALI) Cultures

The air-liquid interface (ALI) model has revolutionized COPD research, providing a closer representation of the lung epithelial environment. Unlike conventional 2D cultures, ALI systems cultivate epithelial cells at the interface between air and liquid, mimicking the physiological conditions of human airways in a 2D system. These models enable the differentiation of polarized epithelial cells into functional types, including ciliated cells, goblet cells, and basal cells, critical for mucociliary clearance and barrier integrity [23,52]. ALI models effectively simulate COPD-related epithelial and inflammatory responses to environmental factors such as cigarette smoke and pollutants. Exposure to cigarette smoke extract (CSE) in ALI systems has been shown to induce the production of pro-inflammatory cytokines like interleukin-6 (IL-6) and Tumor Necrosis Factor Alpha (TNF-α), trigger oxidative stress, and compromise epithelial barrier function, typical hallmarks of COPD pathogenesis [53,54,55]. These findings highlight the relevance of ALI for studying early disease mechanisms, in which epithelial injury initiates inflammatory cascades. Chronic exposure to harmful stimuli drives structural remodeling in the airway epithelium, including goblet cell hyperplasia, basement membrane thickening, and epithelial-to-mesenchymal transition (EMT) [27,55].

ALI models are instrumental to study the impaired repair mechanisms that characterize COPD. Unlike healthy epithelium, which regenerates rapidly post-injury, COPD epithelium exhibits delayed or incomplete repair, leading to persistent damage. ALI cultures provide a controlled environment for examining the role of basal cells and signaling pathways such as Notch and Wnt in epithelial regeneration. This research has elucidated mechanisms of repair failure, informing potential therapeutic strategies [56].

More recently, ALI models have evolved to incorporate multicellular co-cultures, including fibroblasts, endothelial cells, and immune cells such as macrophages or dendritic cells, enhancing the model’s physiological relevance. These advanced ALI models simulate epithelial–mesenchymal and immune–epithelial crosstalk, allowing to investigate of how stromal signals and immune responses shape epithelial remodeling in COPD [57,58]. For example, macrophage-epithelial co-cultures under ALI conditions have shown how proinflammatory polarization amplifies epithelial damage in response to pollutants and smoke [24].

Cell sources significantly influence model behavior. While immortalized cell lines (e.g., BEAS-2B) are frequently used, they exhibit mesenchymal rather than epithelial characteristics and limited differentiation capacity, thus undermining the reproducibility of barrier and secretory phenotypes [59]. Primary human bronchial epithelial cells (HBECs), especially those derived from well-characterized COPD donors, offer superior fidelity in replicating goblet cell hyperplasia, impaired ciliation, and altered cytokine profiles [28,60]. Integration of patient-derived iPSCs and gene-edited cells also enables to explore disease-specific genetic backgrounds and responses to stimuli.

Beyond mechanistic studies, ALI models serve as platforms for drug discovery and evaluation. Their ability to replicate human airway conditions makes them ideal for testing anti-inflammatory drugs, mucolytics, and target therapies for epithelial repair. For example, gene therapy approaches targeting epithelial proliferation and barrier restoration have shown promise in ALI systems, offering potential to mitigate COPD inflammatory and fibrotic effects [61,62]. ALI models are also valuable for studying viral infections that exacerbate COPD, such as those caused by influenza or respiratory syncytial virus (RSV). By replicating compromised epithelial barriers in COPD, ALI systems facilitate research into viral entry mechanisms and potential antiviral interventions, addressing a critical aspect of disease progression [63].

The combination of ALI cultures with organoids, airway-on-chip platforms, and perfusable scaffolds further broadens their application, including biomarker validation, toxicology, and patient-specific therapeutic screening [64]. For example, ALI-organoid hybrids allow longitudinal assessment of epithelial regeneration under cyclic air-liquid exposure, while enabling real-time imaging of mucociliary dynamics [65].

### 2.2. 3D Models

#### 2.2.1. 3D Organoid Cultures

The adoption of 3D models and organoids in COPD research marks a transformative shift from traditional 2D cultures. These systems more accurately replicate the structural and cellular complexity of the lung, and provide a more in-depth understanding of disease mechanisms, including epithelial dysfunction, inflammation, and tissue remodeling [25]. Three-dimensional lung models incorporate epithelial cells, fibroblasts, and immune cells within a matrix that mimics the extracellular environment (Figure 1). This design allows researchers to explore cellular interactions and disease processes, such as how pollutants and cigarette smoke damage the epithelial barrier and drive chronic inflammation. These systems also facilitate studies on immune cell contributions, including neutrophils and macrophages, to the persistent inflammation characteristics of COPD [21,66].

Lung organoids, self-organizing 3D structures derived from stem or progenitor cells, represent a cutting-edge tool for COPD research. Organoids emulate lung architecture and cellular function, encompassing ciliated, goblet, and basal cells. They have been used to study key COPD features, such as impaired epithelial regeneration, mucus hypersecretion, and fibrosis, offering valuable insight into epithelial repair mechanisms and molecular pathways [22,56,67]. A major advantage of 3D models is their ability to accurately simulate the lung microenvironment more effectively than 2D systems. For example, epithelial cells in 3D cultures experience both air and liquid phases, promoting differentiation and barrier function. These models also enable co-cultures with immune and stromal cells, enhancing the study of interactions central to COPD pathogenesis, such as epithelial-immune cell communication and extracellular matrix remodeling [25]. Organoids have proven especially useful in investigating epithelial injury and repair in COPD. Exposure to pollutants or cigarette smoke disrupts epithelial integrity, impairing regeneration. Organoids provide a platform to study growth factors, such as EGFR and TGF-β, and screening therapeutic compounds aimed at promoting epithelial repair or reducing inflammation [67,68]. These models also illuminate the role of immune dysregulation in COPD. In 3D systems, co-cultures with immune cells have revealed how chronic inflammation worsens epithelial damage and tissue remodeling.

Recent scRNA-seq profiling of COPD-derived alveolar organoids has identified disease-specific progenitor dysfunction, including ATII cell subsets with impaired proliferative and regenerative potential [69]. This confirms that the behavior of even in vitro organoids can reflect the clinical status of the donor, suggesting that personalized COPD organoids are a powerful tool for endotyping disease heterogeneity and testing therapies. Recent single-cell atlases of the human lung have provided integrated, consensus-annotated reference datasets covering millions of cells across healthy and diseased states, including COPD [70]. Spatial transcriptomic studies of human lung biopsies have further advanced these insights by mapping where cell types sit and how gene expression varies across tracheal, bronchial, and alveolar regions in both healthy and COPD tissue. These data provide an anatomically grounded molecular reference [71].

Importantly, the origin and differentiation stage of progenitor cells (adult tissue vs. iPSC vs. fetal lung) dramatically impact organoid fidelity and functionality [72]. Therefore, rigorous standardization of protocols, matrix composition, and validation metrics are crucial for reliable COPD modeling and translational applications.

#### 2.2.2. Lung-on-a-Chip

Lung-on-a-chip models have emerged as sophisticated tools in COPD research, replicating the lung complex architecture and dynamic functionality. These microfluidic devices combine epithelial, endothelial, and smooth muscle cells within a controlled environment, mimicking the physiological and mechanical conditions of the human respiratory system (Figure 1). Unlike traditional cell culture models, lung-on-a-chip systems incorporate dynamic processes such as mechanical stretching and fluid flow, crucial for the study of COPD mechanisms [73].

A key feature of lung-on-a-chip models is their ability to reproduce the blood-air barrier and simulate exposure to air and liquid phases to evaluate in vitro the effects of cigarette smoke, pollutants, and viral infections. Studies using lung-on-a-chip systems have demonstrated typical COPD-related changes, including epithelial barrier disruption, increased production of pro-inflammatory cytokines such as IL-6, TNF-α, and IL-8, and elevated oxidative stress markers [74]. These models provide valuable insights into how environmental exposure drives chronic inflammation and structural lung changes [75]. Moreover, lung-on-a-chip devices are helpful in studying the impaired epithelial repair characteristics of COPD. Under normal conditions, the airway epithelium regenerates rapidly after injury. In COPD, however, this process is delayed or dysfunctional, leading to persistent damage and remodeling. Lung-on-a-chip systems facilitate the study of growth factors, extracellular matrix interactions, and stem cell dynamics that influence epithelial repair. This research has highlighted potential therapeutic targets for restoring epithelial integrity and improving lung function [76,77]. These models also serve as platforms for drug testing and evaluation. By accurately simulating COPD pathology, lung-on-a-chip systems allow researchers to assess the efficacy of anti-inflammatory drugs, mucolytics, and experimental therapies targeting epithelial repair and inflammation. Furthermore, lung-on-a-chip devices enable toxicity testing, ensuring novel therapies are both effective and safe [78,79]. The integration of patient-derived cells into lung-on-a-chip systems marks a significant advancement in personalized medicine. Cells obtained from patients, including those derived from induced pluripotent stem cells (iPSCs), can be used to build models that reflect individual genetic and environmental factors influencing COPD progression. These personalized systems facilitate the study of patient-specific responses to therapies, paving the way for tailored treatment strategies [80,81,82]. Despite their promise, lung-on-a-chip models face challenges. Creating systems that incorporate all lung cell types, mechanical forces, and biochemical signals remains complex. While many models effectively replicate core aspects of lung physiology, further refinements are needed to include components such as immune cells, vascular networks, and lymphatic systems for even greater realism. Additionally, the technical expertise and resources required for fabrication and maintenance may limit their accessibility [30,83].

Nizamoglu et al. [22] emphasize that lung-on-a-chip technologies should be embedded within multi-scale experimental pipelines including omics, functional imaging, and machine learning for data integration. These strategies are vital for translating microfluidic outputs into clinically meaningful endpoints, particularly in early drug development and toxicity prediction [84,85].

The use of whole cigarette smoke exposure rather than smoke extract, and the inclusion of breathing motion via cyclic mechanical stretch, has improved model fidelity and increased alignment with in vivo responses, for example, in models demonstrating oxidant-mediated epithelial injury and mucus hypersecretion [86].

Moreover, lung-on-a-chip devices are an interesting platform to investigate comorbidities frequently associated with COPD, such as cardiovascular disease and diabetes. Multi-organ-on-a-chip systems could elucidate interactions between COPD and these conditions, helping identify therapeutic strategies that address systemic effects. For example, combining lung and heart-on-a-chip systems could reveal how COPD-related inflammation impacts cardiovascular health and vice versa. Lung-on-a-chip models offer critical insights into disease mechanisms, drug discovery, and personalized medicine simulating lung physiology and pathology with unprecedented accuracy. These systems will play an increasingly central role in advancing our understanding of COPD and improving treatment outcomes for patients.

#### 2.2.3. Precision-Cut Lung Slices (PCLS) Model

PCLS represent an ex vivo model that retains the native lung architecture, multicellular composition, and physiological microenvironment, offering a bridge between in vitro and in vivo systems in COPD research [48]. PCLS are generated by slicing inflated lung tissue into thin sections (100–500 µm) using a vibratome, preserving alveolar structures, bronchioles, resident immune cells, and extracellular matrix components [87]. This model allows to study airway reactivity, inflammation, fibrosis, and epithelial injury in a near-native context [88]. In COPD research, PCLS have proven especially valuable for investigating the functional consequences of airway remodelling and inflammatory responses. Exposure of PCLS to cigarette smoke, ozone, diesel particles, or pathogens, induces hallmark features of COPD, including goblet cell hyperplasia, mucin overproduction, neutrophilic infiltration, and cytokine release (e.g., IL-8, TNF-α, GM-CSF) [89,90]. Moreover, PCLS have been applied to investigate acute exacerbations of COPD (AECOPD) by mimicking co-exposure to pathogens and environmental pollutants. For example, co-incubation with influenza virus and cigarette smoke extract results in synergistic epithelial damage and barrier breakdown, reproducing in vivo-like exacerbation dynamics [45,91,92]. This capacity to simulate multifactorial insults makes PCLS a relevant model to study host-pathogen interactions, antiviral responses, and drug efficacy. One of the key advantages of PCLS lies in their compatibility with functional assays, including airway contraction, calcium imaging, immunohistochemistry, and multiplex cytokine quantification [88,93]. Furthermore, they can be cultured for several days, allowing longitudinal studies of disease progression and resolution under controlled conditions. However, limitations include restricted viability beyond 5–7 days, donor variability, and limited scalability for high-throughput applications [94]. The integration of PCLS into COPD research pipelines provides a complement to ALI, organoid, and lung-on-a-chip models by offering a native tissue platform for validating cellular and molecular findings. This, in turn, helps to bridge the translational gap between reductionist in vitro systems and complex in vivo models.

#### 2.2.4. Lung ECM-Derived Hydrogels

Lung ECM-derived hydrogels represent an emerging bioengineering platform that aims to more faithfully reproduce the biochemical and mechanical microenvironment of human lung tissue. These hydrogels are generated through decellularization of human or animal lung tissue, followed by enzymatic digestion and gelation, preserving native ECM proteins such as collagen, elastin, fibronectin, and glycosaminoglycans [95,96].The resulting hydrogel mimics the viscoelastic and biochemical cues of the lung matrix, providing a bioactive scaffold that promotes cellular differentiation and functional maturation [66]. In COPD research, lung ECM-derived hydrogels are used to culture airway epithelial cells, fibroblasts, and immune cells under conditions that closely simulate the diseased microenvironment. Notably, hydrogels prepared from decellularized COPD lungs retain pathological ECM signatures: These include altered stiffness and abnormal collagen/elastin composition, enabling disease-specific modeling of fibrosis, inflammation, and epithelial–mesenchymal transition (EMT) [50,51]. These hydrogels support long-term 3D cultures and enable co-culture systems, allowing exploration of cell-ECM and cell-cell interactions that drive COPD progression. Moreover, lung ECM hydrogels facilitate the study of epithelial regeneration under physiologically relevant matrix constraints. For instance, basal cells seeded within ECM-derived hydrogels exhibit context-dependent differentiation into ciliated and secretory lineages. This process is influenced by matrix origin and stiffness [97]. Integration with ALI cultures or microfluidic systems further enhances model complexity, bridging the gap between static 3D matrices and dynamic organ-level models [98].

Recent studies have also employed lung ECM hydrogels for drug screening and personalized medicine applications [99,100]. Patient-derived cells cultured within ECM-matched hydrogels retain disease-specific phenotypes, including mucus hypersecretion, impaired ciliation, and cytokine production. These features make ECM hydrogels a promising tool to evaluate antifibrotic, anti-inflammatory, or regenerative compounds in a COPD relevant environment [51,101]. However, technical challenges remain, including batch-to-batch variability in hydrogel composition, incomplete decellularization, and difficulties standardizing across donors [102,103]. Despite these limitations, lung ECM-derived hydrogels offer a physiologically rich and modular platform that complements organoids, PCLS, and lung-on-a-chip devices in the preclinical modeling of COPD (Table 1).

**Table 1:** Comparative overview of advanced in vitro and ex vivo models for COPD research.

Model	Physiological Fidelity	Reproducibility	Scalability/Throughput	Max Culture Duration	Translational Readiness	Best Suited for (Research Question)
ALI	Moderate-High Polarized pseudostratified epithelium; lacks vascular and mechanical cues [23,39]	Moderate Dependent on cell source; primary HBECs show donor variability; immortalized lines (e.g., BEAS-2B) lack full epithelial fidelity [39,78]	High Compatible with multi-well formats and high-throughput drug screening [45]	Up to 4–6 weeks at ALI	Good Established protocols; used in drug toxicology and regulatory testing [23,46]	Epithelial barrier function; mucociliary clearance; pollutant exposure; viral/bacterial infection; drug toxicology; patient-specific COPD modelling [39,46]
Organoids	High 3D multicellular architecture; multilineage differentiation; lacks ALI and vascular components [47,67]	Low-Moderate Lack of standardized medium composition and matrix protocols; significant inter-laboratory variability [72,104]	Moderate Passageable; increasingly compatible with automated platforms [104]	Weeks/months	Moderate-Good Patient-derived iPSC organoids enable disease-specific drug screening [29,67]	Epithelial regeneration; EMT; mucus hypersecretion; patient-specific drug screening; endotyping COPD heterogeneity [67,104]
Lung-on-a-chip	Very High Integrates epithelium, endothelium, cyclic stretch and airflow; most physiologically complete in vitro model [32,73]	Low Fabrication variability (PDMS-based devices); limited standardization across laboratories [73]	Low One condition per device; not compatible with high-throughput screening [32]	Days/weeks	Promising (early-stage) Useful in drug efficacy and antiviral testing, not yet in regulatory pipelines [32,73]	Biomechanical modelling; host-pathogen interactions; smoke/pollutant exposure under airflow; precision pharmacology; COPD exacerbation dynamics [32,73]
PCLS	Very High Preserves native multicellular architecture, ECM, resident immune cells; closest to in vivo [48,105]	Low High donor variability; inter-laboratory differences in tissue acceptance criteria; no standardized protocol [105]	Low Limited tissue availability; labour-intensive preparation; not amenable to high-throughput formats [105]	5–7 days (standard); up to 14 days with optimized media [105]	Good (validation) Used to bridge in vitro findings and in vivo responses; relevant for ex vivo drug testing [48,49]	Airway reactivity; native inflammation; fibrosis; host-pathogen interactions; ex vivo validation of cellular/molecular findings from reductionist models [48,105]
ECM Hydrogels	High Retains native biochemical and mechanical cues of lung ECM, including disease-specific signatures in COPD tissue [49,50]	Low Batch-to-batch variability in decellularized matrix composition; no standardized protocol across labs [50,51]	Moderate Can be created in multi-well formats; compatible with 3D culture of multiple cell types [51]	Weeks (long-term 3D culture feasible)	Moderate Used in drug screening and personalized medicine; not yet in regulatory frameworks [49,51]	ECM remodelling; fibrosis; epithelial-mesenchymal interactions; cell-ECM signalling; COPD-specific matrix pathology; antifibrotic drug testing [49,50,51]

### 2.3. Critical Limitations of Current 3D Models and Strategies to Address Them

Each platform presents technical and biological limitations that currently hinder their translational potential. These must be weighed against the benefits summarized in Table 1. ALI cultures and organoids lack vascular and mechanical components and heavily depend on the cell source: primary cells vary considerably from donor to donor, while immortalized lines only partly reproduce epithelial differentiation. Conditional reprogramming, defined serum-free media, and iPSC-derived cell banks with agreed differentiation protocols are increasingly being used to make cell sources more reproducible and standardized [39,72]. A further issue for organoids is batch-to-batch variability of animal-derived matrices such as Matrigel, which chemically-defined synthetic hydrogels and standardized matrix formulations aim to solve [104]. Lung-on-a-chip systems are the most physiologically complete, but also the most demanding. Building perfusable microvascular networks is still a hurdle, and the one-condition-per-device format keeps throughput low. Self-assembling endothelial co-cultures, injection-molded or PDMS-free devices, and multiplexed chip arrays are being developed to improve vascularization and scalability, and reduce device-to-device variability [32,73]. PCLS preserve native multicellular architecture but lose viability over prolonged culture and vary widely between donors. Optimized media, controlled oxygenation and minimum reporting standards have extended viability and made results easier to compare across laboratories [105]. ECM-derived hydrogels reproduce disease-specific matrix cues yet are held back by batch-to-batch variability of decellularized tissu, an issue that pooled-donor matrices, standardized decellularization, and hybrid synthetic-ECM scaffolds try to address [50,51]. Before any single approach can be broadly adopted for COPD drug development, several challenges must be addressed, including adding immune components, multi-organ interaction, and agreeing on quality-control metrics. These platforms are complementary rather than interchangeable, and none of them is currently optimal on every axis. Once cell sources are standardized, ALI cultures and organoids offer high reproducibility and throughput, making them well suited for mechanistic studies and medium-scale screening. However, they still lack vascular and immune compartment, which limits their translational reach. Lung-on-a-chip systems provide the most physiologically representative models. They reproduce breathing-like mechanics and the epithelial–endothelial air–blood barrier using patient-derived cells [73]. In addition, perfusable microvascular networks are increasingly built in to improve biological relevance [106]. Their limitations are practical: PDMS devices absorb small molecules and show batch-to-batch variability. Also, the one-condition-per-device format restricts throughput, driving the field toward thermoplastic and multiplexed formats [107]. Consequently, these systems are currently better suited for hypothesis validation than for large-scale screening. PCLS and ECM-derived hydrogels preserve native architecture and disease-specific matrix cues. However, their reliance on donor tissue and decellularization limits inter-laboratory reproducibility [105]. From a translational standpoint, the field is moving towards a fit-for-purpose approach in which platforms are selected according to the biological question rather than ranked hierarchically, while shared reporting standards, defined cell sources, and inter-laboratory validation remain the key prerequisites for regulatory acceptance in COPD drug development.

## 3. Respiratory Infections

### 3.1. Role of Respiratory Infections in COPD

In addition to evaluating the effects of pollutants and/or cigarette smoke, 3D ALI cultures have been used to investigate the role of respiratory infections in exacerbating COPD [108]. While the role of environmental factors and smoking in COPD development is well-known, in recent years it has been understood that infectious pathogens, including viruses and bacteria, can contribute to disease exacerbation and progression. Acute worsening of respiratory symptoms is a hallmark of COPD and significantly impacts on the disease morbidity and mortality. These exacerbations are most triggered by respiratory infections. Patients with COPD are particularly vulnerable to viral and bacterial infections, which can cause acute exacerbations and lead to further lung damage [109,110]. Using 3D lung models at the ALI, researchers can replicate how COPD-affected epithelial cells respond to pathogens, allowing a more accurate simulation of infection dynamics in the disease. For instance, COPD-derived epithelial cells cultured in ALI systems exhibit an exaggerated inflammatory response and increased susceptibility to pathogens like rhinovirus and influenza [111,112]. These findings have important implications for understanding why COPD patients experience more severe and frequent infections, and they highlight the potential of targeting epithelial cell dysfunction to mitigate the effects of respiratory infections in COPD [113].

### 3.2. Respiratory Pathogens

#### 3.2.1. Viruses

Respiratory viruses are major triggers of COPD exacerbations, with rhinoviruses, influenza virus, RSV, and coronaviruses being the most frequently detected [114,115,116,117,118,119,120,121,122]. These infections exacerbate airway inflammation, airflow limitation, and symptom severity [120]. Among them, rhinovirus is particularly prevalent during the colder months and may worsen the disease by promoting IL-6 and TNF-α production, increasing airway inflammation and obstruction [123,124]. COPD patients may also have higher rhinovirus loads and reduced type I and III interferon responses in sputum and lung tissue, potentially impairing antiviral defense [117,125,126]. However, increased IFN-λ1, IFN-λ2, IFN-β, and broader type I and III interferon responses have also been observed in rhinovirus-infected COPD airway epithelial cells in vitro [116,124,127,128]. These apparently conflicting findings may reflect a delayed rather than absent interferon response [129].

Influenza and RSV are strongly associated with severe COPD exacerbations that require hospitalization [130,131,132]. Influenza can also increase susceptibility to secondary bacterial infections, further supporting annual vaccination as an important preventive measure against COPD [131]. Recently, RSV vaccines have been approved [133], and their use is recommended for individuals with chronic respiratory diseases, including COPD. However, their effect on the frequency and severity of COPD exacerbations remains to be fully established and warrants further evaluation [134].

COPD is associated with an increased risk of hospitalization and mortality from COVID-19 [135,136]. Several SARS-CoV-2 vaccines are currently available [137,138,139], and vaccination is expected to reduce the risk of severe outcomes in COPD patients, although its specific effect on COPD exacerbations is not yet fully understood [134]. During the COVID-19 pandemic, masking, social distancing, and infection-control practices were associated with a reduction in severe COPD exacerbations, likely owing to decreased exposure to respiratory pathogens [140].

#### 3.2.2. Bacteria

Several bacterial pathogens are associated with COPD, including *Pseudomonas aeruginosa* [141,142], *Streptococcus pneumoniae* [143,144], *Haemophilus influenzae* [145,146], *Staphylococcus aureus* [147], and *Moraxella catarrhalis* [145,148]. These organisms can colonize the respiratory mucosa even during clinically stable phases of the disease [149]. Hospitalized or mechanically ventilated COPD patients are also vulnerable to secondary infections caused by multidrug-resistant bacteria, particularly *Klebsiella pneumoniae* and *Acinetobacter baumannii*. This vulnerability highlights the potential value of vaccines that reduce infection risk and disease exacerbation [142,149,150,151,152]. The upper respiratory microbiome is dominated by *Moraxella*, *Staphylococcus*, *Corynebacterium*, *Haemophilus*, and *Streptococcus* in the nasal cavity and nasopharynx. *Prevotella*, *Veillonella*, *Streptococcus*, *Leptotrichia*, *Rothia*, *Neisseria*, and *Haemophilus* are instead more abundant in the oropharynx [153]. Although modest microbiome differences have been reported between smokers and nonsmokers across COPD stages [154,155], severe disease is generally associated with reduced bacterial diversity and enrichment of potentially pathogenic species [156,157,158]. This dysbiosis correlates with increased inflammatory mediators, including IL-1β and CXCL8 [157,159,160]. However, the severity of exacerbation may depend more on strain-specific virulence than on the bacterial species alone [161,162,163]. Consistent with this notion, certain *H. influenzae* strains exhibit enhanced epithelial adherence and induce greater CXCL8 secretion [164].

Antibiotics are widely used to manage or prevent severe bacterial COPD exacerbations [165,166,167], but increasing resistance limits their effectiveness. *S. pneumoniae*, *S. aureus*, *H. influenzae*, *P. aeruginosa*, *K. pneumoniae*, and *A. baumannii* are included in the World Health Organization (WHO) 2024 Bacterial Priority Pathogens List, underscoring the urgent need to develop new preventive and therapeutic approaches [168]. Vaccination is recommended when effective vaccines are available. For *S. pneumoniae*, these include 13-, 15-, and 20-valent conjugate vaccines and a 23-valent polysaccharide vaccine, recommended for adults over 65 years of age and for individuals with COPD [134,169]. Although effective in many high-risk populations, protection remains restricted to the serotypes included in each formulation [170]. Studies conducted in Germany [171], Greece [172], Turkey [173], and Thailand [174] nevertheless show low uptake among COPD patients, largely due to doubts on efficacy, limited awareness of benefits, or cost concerns.

No licensed vaccines are currently available against *P. aeruginosa* [175], *S. aureus* [176], non-typeable *H. influenzae* [177,178], *K. pneumoniae* [179], *A. baumannii* [180], or *M. catarrhalis* [178]. While Hib vaccines have proven highly effective against *H. influenzae* type b [181], non-typeable strains remain important drivers of COPD exacerbations [182]. Commercial bacterial lysate preparations containing inactivated strains of *M. catarrhalis*, *S. pneumoniae*, *K. pneumoniae*, *S. aureus*, *H. influenzae*, and other respiratory bacteria are also available [183,184,185,186,187,188,189,190]. These products may provide some protection against exacerbations with few reported adverse effects, but their activity is limited to the strains or serotypes represented, and none of them has received FDA approval [191].

### 3.3. Bacterial and Viral Co-Infections

Bacterial–viral coinfections occur in approximately 25% of patients hospitalized for COPD exacerbations and are associated with greater lung impairment, longer hospital stays, and increased risk of readmission [17,192]. Although some infections may arise independently, respiratory viruses, particularly rhinovirus, can predispose patients to secondary bacterial infections. Experimental rhinovirus infection was indeed followed by bacterial infection in 60% of COPD patients, most commonly involving *H. influenzae* and *S. pneumoniae* [17,193,194]. These episodes may reflect expansion of bacteria already present in the airways, especially *H. influenzae*, rather than acquisition of new strains [195]. This susceptibility may partly result from reduced levels of antibacterial peptides such as secretory leukocyte protease inhibitors and elafin [196]. Rhinovirus can also disrupt epithelial barrier integrity, increase permeability, and promote bacterial adherence by upregulating fibronectin and platelet-activating factor receptors [197]. In addition, degradation of interleukin-1 receptor-associated kinase 1 reduces CXCL8 production and neutrophil recruitment, weakening bacterial clearance [198]. Viral-bacterial coinfection may further amplify airway inflammation by increasing CCL20, human β-defensin-2, and IL-17C expression [199,200,201]. However, COPD patients produce less human β-defensin-2, impairing antibacterial defense, while exhibiting increased IL-17C production that promotes CXCL1 expression and neutrophil chemotaxis [199,200]. This combination of weakened antimicrobial activity and excessive inflammation may contribute to the greater severity of coinfection-associated COPD exacerbations.

### 3.4. 3D Models for Evaluating the Role of Viral and Bacterial Infections in COPD

Because bacterial and viral infections are major drivers of COPD exacerbations, physiologically relevant models are required to investigate the underlying host-pathogen interactions. Conventional 2D cultures fail to reproduce the cellular complexity, tissue organization, or mechanical environment of the lung. However, advanced 3D systems, including lung organoids, ALI cultures, and lung-on-a-chip platforms, provide more representative models of airway infection, epithelial injury, inflammation, and immune responses. Lung organoids reproduce aspects of pulmonary architecture and cellular diversity and have been used to examine pathogen-induced tissue damage and inflammatory responses [30]. Lung-on-a-chip systems additionally incorporate tissue interfaces, fluid flow, and immune-cell circulation. In SARS-CoV-2 models, infection induced cytokine-dependent epithelial innate immune responses and endothelial detachment. Microfluidic airway chips, on the other hand, enabled simultaneous evaluation of antiviral activity, cytokine production, and circulating immune cells during influenza and SARS-CoV-2 infection [31,202]. An alveolus-on-chip containing epithelial and endothelial cells together with macrophages successfully reproduced barrier disruption and endothelial injury following infection with influenza virus, *S. aureus*, or both. These findings support the use of this platform for studying viral–bacterial coinfections, which are relevant to severe COPD exacerbations [203].

ALI cultures generate a differentiated pseudostratified epithelium containing ciliated and mucus-producing cells, making them particularly well suited for investigating microbial adherence, invasion, and viral entry [23]. These models have supported the propagation and study of multiple rhinovirus strains and have been used to examine interactions between *S. aureus* and *P. aeruginosa*, including biofilm formation and epithelial inflammatory responses [204,205,206,207,208].

Although their direct application to COPD-specific exacerbation models remains limited, organoids, ALI cultures, and lung-on-a-chip systems provide valuable platforms for reproducing infection-associated epithelial dysfunction, inflammatory signaling, and polymicrobial interactions. Incorporating COPD-derived cells and disease-relevant environmental conditions into these systems could enhance mechanistic insight and facilitate the development of targeted anti-infective and immunomodulatory therapies.

### 3.5. Drug Discovery and Personalized Medicine

Three-dimensional ALI cultures have shown great promise in drug discovery and personalized medicine. Given the high heterogeneity of COPD, patients display different degrees of airway obstruction and different responses to treatment, therefore making personalized approaches increasingly important. By generating 3D ALI cultures from COPD patient-derived cells, researchers can model individual disease phenotypes and test drug efficacy in a patient-specific manner. This approach allows for the screening of novel therapeutics under conditions that closely resemble the in vivo lung environment This potentially reduces the failure rate of COPD drugs in clinical trials, and improves treatment outcomes for patients [209,210].

The ability to integrate 3D lung models and ALI cultures into high-throughput drug screening platforms is also a promising advancement in COPD research. The basis of conventional drug testing predominantly relies on animal models, which frequently fail to accurately replicate the human lung physiology and the complexity of COPD. Three-dimensional ALI cultures provide a more human-relevant model for testing pharmacological compounds, allowing researchers to evaluate drug effects on epithelial barrier function, mucociliary clearance, and inflammatory responses. Additionally, the ability to culture cells derived from COPD patients in these models facilitates the development of personalized therapeutic approaches. This offers the potential to tailor treatments to the clinical characteristics of individual patients [15,22,87]. Recent advances in this field have brought new insights into potential therapeutic and diagnostic approaches. In particular, probiotics represent a valuable support for COPD treatment. Moreover, the recognition of the pivotal role played by miRNA in diagnostic and therapeutic approaches constitutes a promising field of interest.

A promising avenue for COPD precision medicine is the integration of patient-derived 3D models with high-resolution molecular profiling. Organoids and ALI cultures grown from individual donors can be profiled by single-cell RNA-sequencing and spatial transcriptomics and compared against integrated human lung cell atlases [70,71]. This approach enables the assignment of a donor’s epithelial and progenitor states to distinct COPD endotypes. Adding proteomic, epigenomic, and EV/miRNA data to the same models allows to identify candidate biomarkers in a system where matched patient-derived material is still on hand for functional testing. The emerging endotype-specific signatures can be used to stratify patients and, most importantly, to predict how they will respond to treatment exposing the matched patient-derived model to candidate drugs. This closes the loop between molecular classification and functional validation. AI- and machine learning-assisted analysis [211,212] can transform these large, complex datasets into patient stratification and treatment-prediction tools.

## 4. Emerging Diagnostic Strategies and Therapeutic Approaches for COPD

### 4.1. Probiotics

Probiotics are live bacteria or yeasts that, when consumed in adequate amounts, confer a health benefit on the host. Common probiotic microorganisms include species of *Lactobacillus*, *Bifidobacterium*, and *Saccharomyces* that support gut microbial balance, intestinal barrier integrity, and immune function, and help prevent gastrointestinal disorders and infections [213,214,215,216,217,218,219]. A recent meta-analysis reported that probiotic formulations containing *Streptococcus thermophilus*, *Bifidobacterium longum*, and several *Lactobacillus* species were associated with reduced symptom severity in COPD patients [220]. Intranasal administration of lactobacilli has also shown potential benefits in chronic rhinosinusitis, sinusitis, and chronic rhinoconjunctivitis [221,222,223]. Probiotic strains of *Saccharomyces cerevisiae* may further support epithelial barrier function and modulate immune response by promoting the production of anti-inflammatory cytokines and regulating macrophage and dendritic-cell activity [224,225].

COPD-derived ALI cultures and lung-on-a-chip models could provide valuable platforms for determining whether selected probiotics can reduce pathogen-induced inflammation and improve airway defense mechanisms. These systems enable the assessment of epithelial barrier integrity, cytokine production, immune cell recruitment, pathogen clearance, and tissue repair under physiologically relevant conditions. Probiotics may exert beneficial effects reducing the production of pro-inflammatory cytokines, increasing anti-inflammatory mediators, and preserving epithelial integrity [225]. Studies using these models may help identify strains capable of reducing infection-driven COPD exacerbations. They may also support the development of probiotic-based strategies to reinforce respiratory mucosal immunity.

Despite these encouraging findings, the clinical translation of probiotics for COPD and other chronic respiratory diseases remains complex. Probiotic activity depends on multiple factors, including the specific strain, dose, formulation, route of administration, and disease context. Consequently, results obtained with one species or strain cannot be broadly extrapolated to others [226,227,228]. Clinical studies also substantially differ in design, sample size, intervention duration, baseline microbiome characterization, and outcome measures. This limits direct comparison across studies and complicates regulatory interpretation [229,230]. In respiratory applications, several key questions remain unresolved. These include the optimal route of administration, persistence or engraftment at mucosal sites, interactions with pre-existing airway dysbiosis, and the durability of immunomodulatory effects [227,231]. Safety considerations are particularly important in elderly patients with COPD, individuals with advanced disease, and patients with frequent exposure to antibiotics, corticosteroids, impaired epithelial barriers, or other forms of immunosuppression [226,232]. Potential risks include product contamination, transfer of antimicrobial-resistance determinants, inappropriate immune modulation, and, in rare cases, invasive infections [226,232,233,234]. Future studies should integrate mechanistic testing in COPD-derived ALI cultures and lung-on-a-chip systems with adequately powered, strain-specific clinical trials. Standardized manufacturing and viability controls, genomic safety assessment, and rigorous monitoring of adverse—events will also be essential. Together, these measures are needed before probiotic-based respiratory interventions can be advanced toward routine clinical use [226,230,232,233].

### 4.2. Extracellular Vesicles and miRNAs in COPD Diagnosis

**Figure 2:**
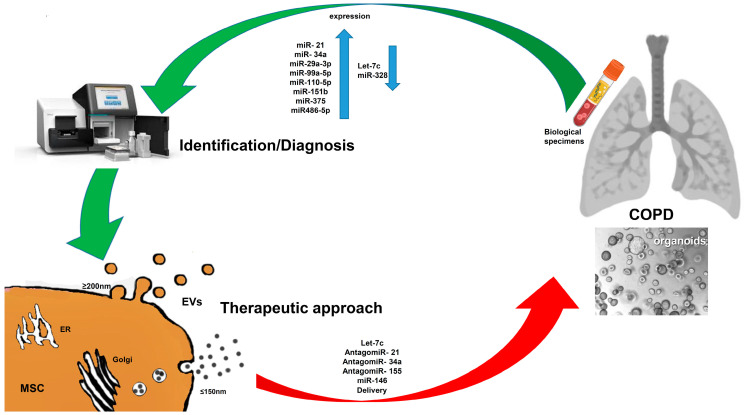
Emerging strategies in diagnosis and potential therapeutic approaches for COPD treatment. Biological samples from patients and in vitro models (organoids) were analyzed to detect differentially expressed miRNAs. The identified miRNAs were evaluated in in vitro models to reduce the effect of the disease. EVs from MSCs or other sources as a useful tool for miRNAs or AntagomiRNA delivery and therefore a potential therapeutic strategy.

The challenging clarification of COPD onset mechanisms including host-pathogen interactions, and in vitro and in vivo models have opened new perspectives on how to integrate functional studies with biomarker discovery and therapeutic innovation. In this regard, EVs and miRNA have emerged as active modulators of inflammation, tissue remodeling, and immune responses, while also representing attractive non-invasive diagnostic biomarkers for early COPD detection [235,236,237]. EV/miRNA-based molecular profiling with 3D patient-derived cultures may ultimately support the development of personalized therapeutic strategies and improve disease stratification [237] (Figure 2).

MiRNAs are a class of small non-coding RNAs (19–22 nucleotides) that regulate gene expression by binding to the 3′ untranslated region of mRNA. One single miRNA specifically inhibits the translation of different genes through its specific binding. These small RNAs play a fundamental role in several normal and pathological processes, from organ development [238] to neoplastic transformation [239]. In 2024, Ambros and Ruvkun were awarded the Nobel Prize in Physiology or Medicine for discovering and describing miRNAs and the remarkable impact of these regulating factors [240]. Furthermore, it has been observed that miRNAs represent a powerful instrument for diagnostics [239]. In particular, it has been demonstrated that EVs can carry different miRNAs through circulating body fluids [239]. As a result, numerous research groups have focused on the characterization of specific circulating miRNAs for disease prognosis and staging [241,242], as they can be readily detected and analyzed in a range of biological fluids [239]. At this regard, it has been demonstrated that air pollution and cigarette smoke inhalation can affect EV and miRNA release, not only in serum [243], but also in bronchial alveolar lavage fluid (BALF) [19] and breastfeeding milk [244]. The release of EVs mediates intercellular communication, carrying miRNAs that can modulate inflammatory response [245,246]. The opportunity to establish the specific signature of miRNA EVs to define COPD pathological onset has prompted diverse groups to analyze various body fluids [19,243,247,248]. One example is the exhaled breath condensate (EBC), recently revised by Kita et al. [249]. With this method, some authors have found a reduction of miR-328 and miR-21 in COPD patients versus healthy subjects [250]. Bronchoalveolar lavage (BAL) has been used to evaluate, by RT-PCR, the expression of specific miRNAs (hsa-miR-223-5p, 16-5p, 20a-5p, 17-5p, 34a-5p, and 106a-5p) in COPD and other lung diseases, such as non-small cell lung cancer (NSCLC), which can be considered an end stage of the disease [251]. This analysis was carried out in a small cohort of patients [251], allowing to identify characteristic expression changes of some miRNAs (Figure 2). In particular, miR-34a was clearly upregulated in patients with COPD, not only compared to normal samples, but also to NSCLC [251]. This miRNA is involved in many processes, ranging from proliferation inhibition [252] to tumor suppression [253]. Similarly, a recent study promoted by the Karolinska Institute, Clinical & Systems Medicine Investigations of Smoking-related Chronic Obstructive Pulmonary Disease (COSMIC), examined COPD development in 120 smoking and non-smoking patients. The study analized numerous factors, from anamnesis to molecular aspects, in order to discover specific diagnostic COPD markers [254]. Microarray analysis in BAL-derived exosomes has revealed the upregulation of the miR-29 family in COPD patients [254] (Figure 2). Recently, Sundar et al. identified miR-29a-3p through NGS analysis of the miRNA pattern in EVs isolated from the serum of smokers and non-smokers [255]. The NGS results showed a pattern of 15 miRNAs expressed in common in EVs from smokers and COPD patients [255]. Some of these (the most abundant based on their read counts) were validated on bronchial epithelial cells (BEAS-2B) treated with 0.5% of cigarette smoke extract (CSE). RT-PCR analysis revealed that miR-29 a-3p is upregulated in EVs derived from CSE-treated BEAS [255]. The same result was observed in other miRNAs (miR-99a-5p, miE-110-5p, miR-151b, miR-375, miR-486-5p), in particular let-7a, let-7i-5p. The association of let-7 with COPD and lung cancer has been demonstrated in various reports [256,257]. In particular, a clear reduction of let-7c in the sputum of COPD smokers was observed by RT-PCR [258] (Figure 2). Similarly, in other studies, the serum level of let-7 in COPD patients is reduced, as assessed by RT-PCR [259] (Figure 2). This reduction has been associated with an increase in IL-6, considering the direct action of let-7 in this cytokine expression [259,260]. This result is consistent with the observation in mouse models exposed to cigarette smoke (CS), where the treatment induced an augmented expression of IL-6 and a reduction of let-7, associated with airway remodeling and increased inflammation. In particular, the effect of let-7 on IL-6 expression was correlated with macrophage M2 polarization in the alveoli of COPD patients and mouse models [261]. Macrophage polarization was also correlated with miR-21 expression in COPD mouse models [262]. In particular, CS and CSE-exposed mice showed increased levels of miR-21, which were correlated with macrophage polarization [262]. As a result, miR-21 inhibition reduced macrophage transformation to the M2 phenotype, diminishing lung damage [262]. Recently, the potential effect of miR-21 was also linked to the regulation of phospholipase and tensin homolog (PTEN) [263]. Its expression is regulated by the direct binding to the mRNA 3′UTR, allowing modulation of the PTEN/AKT/NF-κB axis [263]. The consequent evaluation of lung tissues from COPD patients displayed an increased expression of miR-21, and a reduction of PTEN in contrast to non-smokers [263] (Figure 2). Furthermore, downstream pathway analysis in COPD patients showed an increase of pAKT and p-NF-kB in association with PTEN reduction [263]. These results corroborate the observation of serum miR-21 increase, associated with the early pathogenic COPD onset in asymptomatic heavy smokers [264]. The high level of circulating miR-21 has also been correlated with a significant increase in mortality in younger hospitalized COVID-19 patients [265].

Despite the limitations of this indication, miR-21 could represent an interesting circulating marker in patients in association with clinical parameters [264]. However, the integration of multi-network modeling is essential to correlate the diverse layers of gene regulatory networks and improve our classification of the biological mechanisms stratifying COPD [211]. A machine learning approach has recently been developed in this field as diagnostic support, based on circulating miRNA classification [266]. Given the crucial role of miRNAs in genetic expression regulation, several studies have demonstrated that these small molecules are a useful tool for diagnosis and staging of numerous diseases, including COPD (Table 2). Artificial intelligence (AI)-assisted analysis goes beyond classifying miRNAs. Open-source digital-pathology platforms offer extensible tools for automated batch processing, cell detection and quantitative classification on histological sections [212] These are becoming an effective tool to characterize the phenotypes of tissue-based experimental systems. Integrative multi-network approaches combining gene-expression similarity, protein-protein interactions, transcription-factor regulons, and validated miRNA interactions, have also been used to pinpoint the biological functions altered in specific COPD conditions [211]. Further enrichment of data generated from 3D models could facilitate the development of AI-assisted platforms for multi-omic analysis and patient stratification.

For circulating miRNAs, the main barrier to clinical implementation in COPD is variability before and during measurement. Blood collection, hemolysis, centrifugation, RNA extraction, and the quantification platform can all influence the results. Also, the lack of an agreed normalization strategy makes signatures hard to reproduce across studies [267,268]. Consequently, no validated cut-offs or robust inter-laboratory signatures are currently available, representing a major obstacle to the routine use of miRNA assays [269]. Reliable diagnostic use will require harmonized reporting, standard operating procedures, and adequately powered validation cohorts.

### 4.3. EV and miRNA Therapeutic Perspective

Following their identification as circulating diagnostic signatures, EVs and miRNAs have emerged as potential therapeutic agents for COPD due to their ability to influence different molecular pathways. The dualistic diagnostic and therapeutic role of these molecules is important: the recent evaluation of their effect in 3D in vitro platforms allows mechanistic validation and targeted delivery studies.

Current therapeutic treatment of COPD relies mainly on three classes of molecules: beta-2-agonists, including short-acting (SABA) and long-acting (LABA); long-acting muscarinic antagonists (LAMA); and inhaled corticosteroids (ICS) [270]. However, all these approaches have significant limitations. First, these drugs are primarily aimed at reducing the symptoms, rather than slowing down or halting disease progression. Moreover, their use is associated with a variety of side effects, primarily cardiovascular events [271]. Another significant limitation of COPD therapy arises from the branched structure of the respiratory system, which affects the successful delivery of drugs to the lower airways (bronchi, bronchioles, alveoli), where drug absorption typically occurs more efficiently [272]. In order to overcome these challenges, the scientific community has progressed on two different fronts: enhancing drug delivery through optimized carrier dimensions, and identifying new therapeutic targets. In this context, nanoparticle (NP) delivery systems have emerged as a promising strategy to improve drug delivery and anti-inflammatory effects. Two different research groups demonstrated that NPs can promote anti-inflammatory cytokine expression in in vitro COPD model [273], and mitigate inflammation and oxidative stress in a human in vitro model exposed to cigarette smoke [274]. This approach led to growing interest in the dual diagnostic-therapeutic role of EVs and miRNAs, particularly when studied in conjunction with advanced 3D in vitro platforms such as ALI cultures and lung-on-a-chip systems [275]. These models offer physiologically relevant environments in which to validate the functional impact of these molecules, and assess targeted delivery approaches. Alongside the use of biological drugs, which unfortunately are not always effective, research into new therapeutic treatments for COPD is making significant efforts to improve drug distribution.

Drug adsorption is associated with intrinsic physical and chemical particle properties, and with specific patient features, including disease stage [272]. Recently, in this context, Zimmermann et al. proposed a spray-dried microparticle system consisting of lipid nanoparticles with encapsulated siRNA to work as a new siRNA-based therapy system for respiratory diseases, including COPD [276]. Many years ago, the efficacy of nano-based delivery was exploited to specifically target corticosteroids, and attempt to reduce systemic side effects [277]. Given the opportunity offered by nanoparticle systems, Zoulikha et al. proposed a specific polymeric nanoparticle for miR-146a delivery [278]. In particular, it was demonstrated that this miRNA, adsorbed into this nanoparticle, showed a continuous release after 24 h and exerted a dual biological function. There is in vitro evidence that miR146a reduces the gene expression of Interleukin 1 Receptor Associated Kinase 1 (IRAK1) playing a key role in triggering the innate immune response versus external microbiological attacks. Moreover, the in vitro activity of the IL-8-promoter shows a potential anti-inflammatory effect for COPD treatment [278] (Figure 2).

Mesenchymal stromal cell (MSC)-derived EVs represent a unique tool for carrying drugs, nucleic acids (DNA, RNA) or proteins [279,280,281]. Their use has been investigated in various pulmonary diseases [135,239,282,283,284]. MSCs have immunomodulatory characteristics, connected to their secretome [285]. As a result, MSCs of various sources have been investigated and tested with different methods to enhance their therapeutic potential [286,287,288,289] (Figure 2).

A recent study demonstrated that EVs derived from umbilical cord MSCs in a murine model of cigarette smoke-induced COPD 3D organoid culture showed an anti-inflammatory and immunomodulatory action on epithelial regeneration and alveolar repair in lung epithelial progenitor derived cells [290].

Baker et al. identified miR-570-3p as a novel regulator of cellular senescence and inflammation [291] and found elevated levels of this miRNA in samples from COPD patients (tissues, epithelial or mononuclear cells), alongside altered senescence markers. These included reduced gene expression of anti-aging genes sirt1 and cdk4, in addition to elevated p21 and the senescence-associated secretory phenotype (SASP) proteins, MMP-9 and CXCL8 [291]. Baker et al. also found that miR-570-3p inhibition, through a specific antagomir, can suppress senescence, and reduce pro-inflammatory release [291]. Almost ten years ago, another team of researchers demonstrated that miR34a could also represent an interesting therapeutic target [292]. In particular, they observed that antagomir-mediated miR34a inhibition can counteract the overexpression of this miRNAand, consequently, restore SIRTUIN-1 and -6 mRNA levels, both codifying for anti-aging proteins [292].

Numerous miRNAs have been identified as relevant in COPD pathogenesis, because of their involvement in inflammation, vascular or airway remodeling, mucociliary clearance, and epithelial-mesenchymal transition. Another potential therapeutic target for inhibition is mir155, related to inflammation and lung function parameters of airflow limitation [293]. Indeed, the administration of miR-155 inhibitors in the lungs of a COPD mouse model exposed to cigarette smoke led to a reduction of pro-inflammatory immune cells [293] (Figure 2).

Some miRNAs represent promising therapeutic agents for other diseases (e.g., colon, ovarian or breast cancer) and therefore could have potential applications in the context of COPD (Table 1), in particular miR21 and miR145. The latter, for example, has a controversial role: it has shown a protective action in human bronchial epithelial cells by inhibiting apoptosis and inflammation [294]. However, another study focused on the link between miR145 and cystic fibrosis transmembrane conductance regulator (CFTR) expression [295]. One of the features of COPD is CFTR dysfunction and, consequently, mucociliary clearance impairment [296,297]. Given this scenario, Dutta et al. demonstrated the important involvement of miR145 for CFTR suppression induced by cigarette smoke and that inhibition of miR145 using antagomir in an in vitro model can restore the expression of both CFTR and another important chloride channel [295] (Figure 2).

In the context of altered miRNA inhibition in COPD, a recent study conducted a microarray analysis of the miRNA expression pattern in the lungs of a COPD mouse model [298]. This in-depth molecular analysis highlighted that miR-21 is the second highest up-regulated miRNA, both in lung epithelium and macrophages [298]. The inhibition through a specific miR-21 inhibitor induced a decrease in the pro-inflammatory cell recruitment in the airways and was associated with improved lung function and NF-kB activity [298] (Figure 2).

The effect of miR-21 inhibition was also demonstrated on macrophage polarization in COPD mouse models, where it can stimulate the discovery of promising applications to therapeutic intervention [262]. The inhibition of this miRNA in knockout mice can impact the occurrence of emphysema by modifying M1/M2 polarization [262]. In particular, it has been observed that the M2 reduction caused by miR-21 inhibition induces a clear change in TNF-α expression [262]. It has also been observed that adipocyte-derived EV miR-34a inhibits M2 macrophage polarization [299], and prompts an M2 to M1 switch in NSCLC [300,301,302]. EVs have been found to carry miR-34 to target cells [253] for therapeutic purposes in neoplastic disease, as well as being evaluated in two clinical trials (NCT02862145, NCT01829971). Unfortunately, to date, no studies have been attempted in COPD models or patients. In this regard, a recent study evaluated the use of inulin-stabilized EVs to produce an inhalable dry powder [303]. This compound was tested on lung organoid models, demonstrating that EVs significantly enhance organoid formation. The main goal of this system is the preservation method that enables handling EVs at room temperature without compromising their stability [303]. These advances could pave the way for the development of a spray formulation with convenient administration potential.

**Table 2:** miRNA-COPD Association in Therapeutic and Diagnostic Studies.

*miRNAs*	Involvement in Lung Disease	Study Model	Clinical Impact	References
**let-7c**	Reduction associated with increased expression of Tumor Necrosis Factor Receptor-2 (TNFR2)	Sputum and bronchial epithelial cells from severe COPD smoking patients	Diagnostic marker	[258]
	Down-regulation associated with IL-6 increased expression	Brochial epithelial cells from COPD smoking patients; in vitro model of CS-exposed HBE; peripheral lung tissue of CS-exposed mice	Diagnostic marker	[259]
	Decreased expression induced by CS treatment promotes increased release of IL-6, acting on alveolar macrophages and inducing polarization of M2 macrophages responsible for COPD emphysema through MMPs release	Human lung tissue and serum; lung tissue of CS-exposed mice	Therapeutic target to slow COPD	[261]
**miR-21**	Increased expression of this miRNA induced by CS promoting macrophage M2 phenotype	In vitro RAW264.7 cells CS-exposed; lung tissue and bone marrow-derived macrophages isolated from COPD mice	Gene targeting to inhibit M2 macrophages transformation	[262]
	Associated with early pathogenic COPD onset in asymptomatic heavy smokers. Increased in COPD smokers, associated with PTEN reduction, and consequent increase of pAKT and p-NF-kB	Peripheral lung tissue of COPD patients; in vivo CS-exposed mice; in vitro CS-exposed HBE	Its inhibition reduces inflammation	[263]
	Increased levels associated with early pathogenic COPD onset in asymptomatic heavy smokers	In vivo mouse model; patient serum (healthy, symptomatic heavy smokers, COPD heavy smokers)	Predictive marker for COPD development in heavy smokers	[264]
**miR-29 family**	Upregulated in exosomes (COSMIC study)	COPD BAL samples	Diagnostic marker	[254]
**miR-29 a-3p**	Upregulated in EVs derived from BEAS treated with CSE. EV miRNAs are novel circulating pulmonary disease biomarkers	EVs derived from plasma of nonsmokers, smokers and COPD patients; human bronchial epithelial cells exposed to CS	Strong translational potential to develop biomarkers for diagnosis, prognosis, and therapeutic treatment of COPD	[255]
**miR-34a**	Upregulated in bronchoalveolar lavage (BAL), and involved in proliferation inhibition and tumor suppression	Lung samples from cohort of NSCLC and COPD patients	Diagnostic marker	[251]
**miR-328**	Reduced in COPD, and targeting IL-13, IL-5, IL-1b, IL-8, TLR2	Exhaled Breath Condensate (EBC)	Inflammation profiling using EBC as noninvasive source of COPD biomarkers	[250]
**miR21**	Up-regulated miRNA in airway epithelium and lung macrophages and correlated with reduced lung function in COPD	Lungs of mice with COPD induced by CS treatment	Therapeutic use of specific mir-21 inhibitor to decrease pro-inflammatory cell recruitment in airways, and improve lung function	[298]
**miR34a**	Overexpression of miR-34a mimic responsible for reduced SIRT1/-6 (mRNA and protein)	Lung tissue and sputum from COPD patients	Atagomir inhibition to reduce overexpression and restore anti-aging proteins SIRTUIN-1 and -6, usually downregulated in COPD patients	[292]
**miR145**	Upregulated from TGF-β and CS, capable of suppressing CFTR	Human bronchial epithelial cells	Antagomir inhibition restores expression of CFTR and other chloride channels and exherts protective action inhibiting apoptosis and inflammation	[295]
**mir155**	Increased expression of miR-155 targets	Lung tissue of healthy smokers and COPD patients; lung tissue and alveolar macrophages of in vivo mouse model after CS-treatment; wild type and KO mice model CS-exposed to quantify inflammatory cells, chemokines and cytokines in BAL fluid and lung tissue	New therapeutic target in COPD to reduce pro-inflammatory immune cell levels	[293]
**miR-570-3p**	Elevated levels in inflammation and reduced anti-aging genes sirt1 and cdk4, elevated proteins p21, MMP-9, and CXCL8	Epithelial or mononuclear cells of COPD patients	Antagomir inhibition suppresses senescence and reduces pro-inflammatory cytokine release	[291]

## 5. Conclusions

The adoption of advanced 3D in vitro models, including ALI cultures, lung organoids, and lung-on-a-chip platforms has significantly enhanced our ability to investigate the complex pathophysiological mechanisms underlying COPD. These systems provide a physiologically relevant environment that closely mimics the human lung microarchitecture. As a result, they enable the study of epithelial dysfunction, tissue remodeling, immune dysregulation, and host-pathogen interactions under dynamic and controlled conditions.

Most importantly, the integration of 3D models with emerging biomolecular approaches, such as EVs and miRNAs, is opening new perspectives for both diagnostic and therapeutic applications. Circulating EV-associated miRNAs have shown promise as non-invasive biomarkers for early COPD detection, disease staging, and phenotypic stratification. At the same time, numerous miRNAs have demonstrated regulatory functions in inflammation, epithelial regeneration, and aging pathways, highlighting their potential as targeted therapeutic interventions. Their delivery through engineered EVs or nanoparticle-based systems may further enhance their clinical potential.

Moreover, 3D in vitro models provide an essential translational platform to validate the mechanistic roles of miRNAs and EVs, assess their delivery efficacy, and advance personalized medicine approaches. Probiotic-based strategies also emerge as promising adjunct therapies. Their potential can be explored in dynamic co-culture systems that replicate airway infections and immune interactions.

A further evolution of these 3D models is represented by multiorgan-on-chip systems. These platforms enable a more precise evaluation of this complex pathology and facilitate the standardization of treatment strategies in high-throughput screening, while overcoming the limitations of in vivo models. A complementary direction is the integration of patient-derived 3D models with multi-omics profiling and AI. Patient-derived ALI cultures, organoids, and lung-on-a-chip systems can generate individualized epithelial and immune signatures that recapitulate patient-specific inflammatory and treatment response phenotypes in COPD and asthma [107]. When combined with single-cell and spatial multi-omics, these platforms generate high-dimensional datasets that require advanced computational integration. In this context, AI and machine-learning methods offer scalable tools to interpret imaging data, integrate multi-omic layers, and guide experimental workflows. These approaches link mechanistic in vitro readouts with patient stratification [304]. Moreover, the integration of AI with bioinformatics and multi-omics analyses is creating an intelligent framework for COPD research and management, spanning the continuum from early risk prediction to phenotype-driven therapeutic decisions [305]. The convergence of patient-derived organ-on-chip technologies, multi-omics, and AI is therefore expected to accelerate the transition from descriptive disease modeling to predictive, patient-centered precision medicine in COPD.

Despite the promising therapeutic potential of EVs in COPD, their clinical translation remains limited by the lack of standardized procedures for EV preparation, characterization, and quality control. Addressing these issues is essential for the development of reliable cell-free therapies [306]. Standardization is particularly challenging because EV properties can vary according to their cellular source, including Wharton’s jelly-derived MSCs, dermal MSCs, bone marrow-derived MSCs, and umbilical cord-derived MSCs [299,300,301,302,303,304]. This variability has important implications for potency, reproducibility, safety, and regulatory acacceptance. In this context, the implementation of robust GMP-compliant manufacturing procedures is critical for the development of EV-based advanced therapy medicinal products [306,307,308,309,310,311]. At present, establishing suitable GMP processes for clinical-grade EV preparations remains a major challenge [307,310,311,312]. Nevertheless, despite these limitations, cell-free EV-based strategies represent a promising future approach to improve therapeutic intervention in patients with COPD.

In conclusion, the integration of 3D lung models with molecular and cellular innovations is driving more precise, predictive, and patient-centered approaches to COPD research and treatment. Future studies should focus on validating these strategies in clinically relevant settings to accelerate their translation into effective diagnostic and therapeutic tools.

## Data Availability

No new data were created or analyzed in this study. Data sharing is not applicable to this article.
